# Structural insights into the catalytic cycle of G protein–coupled receptor kinase 5 and a possible regulatory site for potassium ion

**DOI:** 10.1016/j.jbc.2025.110309

**Published:** 2025-05-29

**Authors:** Yueyi Chen, John J.G. Tesmer

**Affiliations:** 1 Department of Biological Sciences, Purdue University, West Lafayette, Indiana, USA; 2 Department of Medicinal Chemistry and Molecular Pharmacology, Purdue University, West Lafayette, Indiana, USA

**Keywords:** catalytic cycle, enzyme conformation, G protein–coupled receptor, G protein–coupled receptor kinase, GRK5, phosphorylation, potassium ion, protein kinase, sangivamycin, serine–threonine kinase, structural biology, X-ray crystallography

## Abstract

G protein-coupled receptor (GPCR) kinases (GRKs) instigateGPCR desensitization, but despite many available structures, a molecular understanding of their function and catalytic cycle remains incomplete. We present six GRK5 crystal structures that capture both open and closed states of its kinase domain as well as complexes with the ligands sangivamycin (Sgv), an adenosine analog, and ATP. The Sgv-bound structure is distinct from the previously reported GRK5·Sgv structure and features an ordered N-terminal helix that docks to the kinase hinge, mimicking its interactions in GPCR or Ca^2+^·calmodulin-bound GRK complexes. GRK5 undergoes a dramatic conformational change in the crystals to a ligand-free, open state with a disordered N terminus when K^+^ is omitted from the harvesting solution. This transition to a ligand-free structure, not structurally observed for the GRK4 subfamily, most likely occurs through the release of the K^+^ ion from its binding site close to the kinase domain hinge in the Sgv-bound complex. Two structures of GRK5 in complex with Mg^2+^ and Mn^2+^·ATP were obtained *via* soaking crystals of the open state, which we hypothesize are reflective of a substrate-loading stage. Although K^+^ significantly stabilizes GRK5 in its closed, near-active conformation, potassium citrate and KCl inhibit kinase activity just as potently as sodium citrate and NaCl, respectively, suggesting that K^+^ traps a closed conformation compatible with Sgv–AMP but incompatible with ATP, thereby inhibiting the catalytic cycle. Thus, changes in K^+^ concentration could play a regulatory role for GRK5 in scenarios where activated GPCRs are coupled to G protein–responsive potassium channels.

G protein–coupled receptor (GPCR) kinases (GRKs) are Ser–Thr kinases well known for their roles in regulating GPCR signaling through phosphorylation-induced receptor desensitization. Seven GRK isoforms (GRK1–7) distribute differently among tissues, with GRK1– and 7 found in the retina, GRK4 predominantly in testes, GRK6 in neuronal and immune cells, and GRK2, 3, and 5 broadly expressed in many different tisssues cellty (1, 2). GRK2 and GRK5 are the most abundant isoforms in cardiomyocytes, and they are involved in the progression of heart failure by contributing to maladaptive cardiac remodeling (3, 4). Furthermore, upon binding Ca^2+^·calmodulin (CaM), GRK5 becomes activated and enters the nucleus where it regulates hypertrophy by phosphorylating histone deacetylase 5, thereby promoting the expression of hypertrophy-associated genes through transcriptional factor myocyte enhancer factor 2 (5, 6, 7).

All GRKs share a similar domain architecture consisting of a kinase domain (small and large lobes) inserted into a loop of a regulator of G protein signaling homology (RH) domain. GRK2 and GRK3 in addition contain a C-terminal Pleckstrin homology domain that recognizes G_βγ_ subunits. In the GRK4 subfamily (GRK4–6), the main building blocks are the small lobe (or “N” lobe) consisting of three discontinuous segments (residues 1–32, 180–270, and 490–510 in human GRK5), the large lobe (or “C lobe”: residues 271–450), and the RH domain (residues 33–179 and 511–590) (Fig. 1*A*). Because GRKs belong to the AGC kinase family (represented by protein kinases A, G, and C), their catalytic domains contain highly conserved regulatory features (2, 8, 9) including an element called the kinase C-tail, which spans both lobes of the kinase domain (10). The AGC kinase C-tail has three components: the C-lobe tether, which binds the large lobe, the active site tether (AST) that forms part of the ATP-binding site, and the N-lobe tether that contributes to the small lobe (Fig. 1*B*) (10). Unlike most AGC kinases, GRKs are not phosphorylated in their activation loop as part of their regulation (although some are at secondary sites in their kinase C-tail), and their “DLG motif” has so far always been observed in the “in” (presumedly active) conformation (11). Despite this, their basal activities are very low, allowing them to be enhanced by orders of magnitude when they engage activated GPCRs (12).

**Figure 1:**
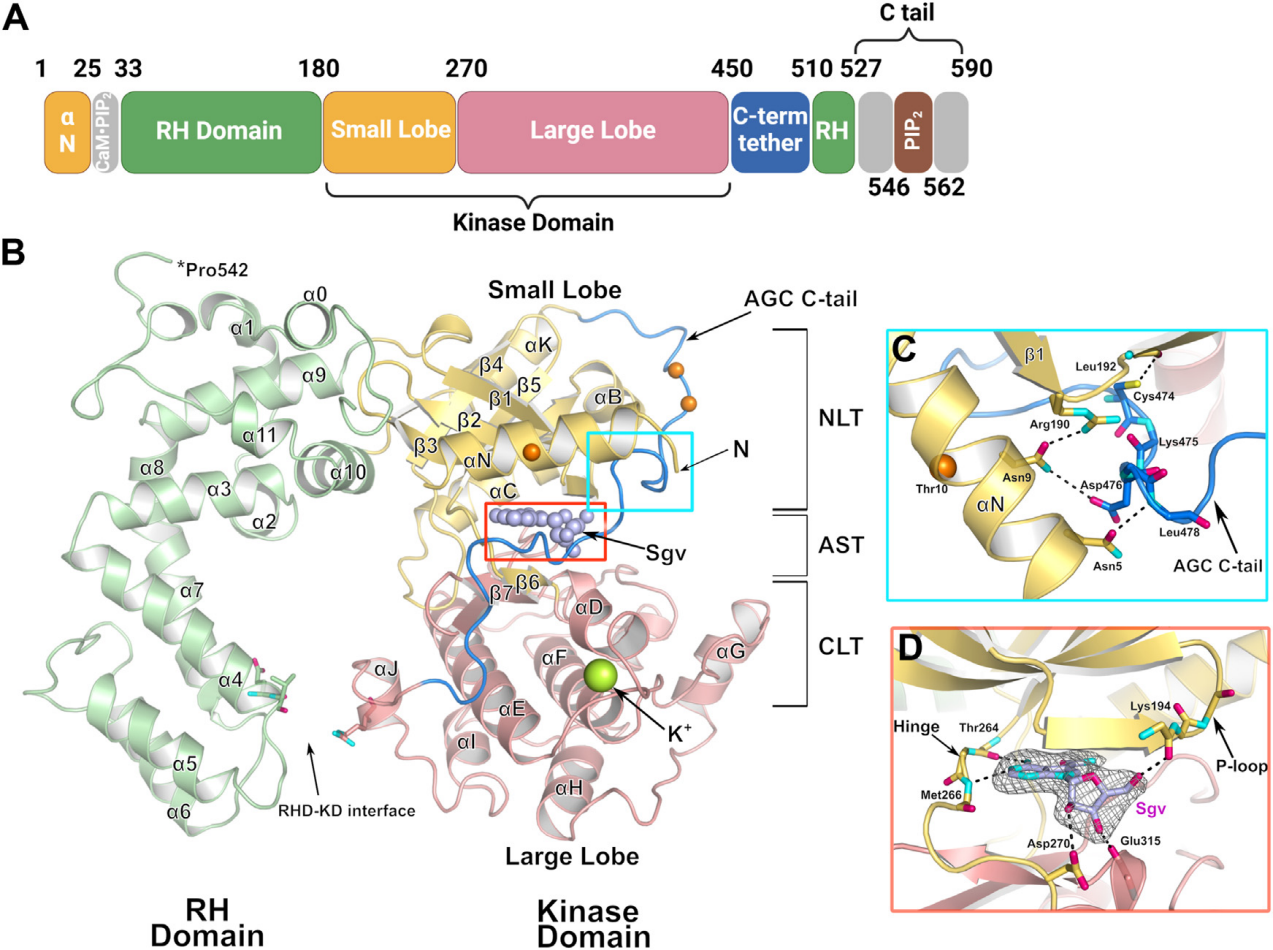
**Domain architecture of GRK5 and its complex with Sgv.***A*, domains of full-length WT human GRK5 from residue 1 to 590, encompassing the RH domain, kinase small lobe, kinase large lobe, and AGC C-tail. The C-tail is further subdivided into the C-lobe tether (CLT), active site tether (AST), and N-lobe tether (NLT) (10). *B*, GRK5·Sgv adopts a closed conformation that binds K^+^. Sgv is shown with *light purple spheres* and K^+^ as a *bright green sphere*. Residues undergoing phosphorylation identified by mass spectrometry were demarked by *orange spheres*. Residues contributing to the RH domain–kinase large lobe interface (RHD–KD) are shown in *sticks*. *C*, bridging interactions between residues in αN, the small lobe, and the CLT. Hydrogen bonds are shown as *black dashes*. *D*, interactions of Sgv (*light purple carbons*) within the adenosine-binding site of GRK5. The Polder omit map (*gray mesh*) for Sgv was contoured at 5 σ. GRK5, G protein–coupled receptor kinase 5; Sgv, sangivamycin.

Previous studies of the GRK–GPCR complexes support an important role for the GRK N-terminal α helix (αN) for engaging the receptor at one end, whereas engaging the hinge and AST region of the kinase domain on the other end to stabilize the closed and active conformation (13, 14). A similar configuration for αN has been observed in GRK5 and GRK6 complexes with the adenosine analog sangivamycin (Sgv), wherein αN packs against Ca^2+^·CaM (15) or a protein lattice contact (12), respectively, *in lieu* of an active GPCR. Another GRK5 structure bound to a small-molecule inhibitor exhibited an intermediate closed conformation but without an ordered N terminus (16). In other structures of GRK5 bound to Sgv and the ATP analog adenylyl-imidodiphosphate (AMP-PNP) (17), and of GRK5 in bound to a small-molecule inhibitor based on sunitinib (18), an open configuration is observed. What the Sgv-bound, closed state of GRK5 represents physiologically is not known. For example, we note that neither GRK5 nor GRK6 could be crystallized in an Sgv-like state when one substitutes ATP for Sgv, indicating that the conformation when bound to Sgv has fundamental differences from the ATP-bound state. It is also unknown whether the kinase domain of GRKs cycles between open and closed states during multiple rounds of catalysis on a GPCR. Bridging these gaps in knowledge is important for a complete understanding of the GRK catalytic cycle.

Here, we introduce six new crystal structures of human GRK5, which provide additional insight into these questions and suggest another potential physiological regulator of GRK5. We solved three structures of GRK5 bound to Sgv: WT GRK5 (heterogeneously phosphorylated), GRK5_D311N_ (catalytically inactive and homogeneous), and WT GRK5 at low pH. All three adopted a relatively closed conformation. A ligand-free GRK5 structure was also obtained when harvesting these crystals in the absence of K^+^ and adopted an open conformation, allowing us to also obtain ligand complexes by soaking these crystals with ATP and Mg^2+^^/^Mn^2+^ ions. Overall, the data support a model wherein the catalytic domain transitions between open and closed states to reload substrates and release products but only while bound to an activating protein such as a GPCR (12, 13, 14). Binding and release of the αN helix to the kinase domain is a key part of the cycle that allows domain closure and couples catalytic cycle progression to sensing an activated GPCR.

## Results

### Structures of GRK5 in complex with Sgv

**Figure S1:**
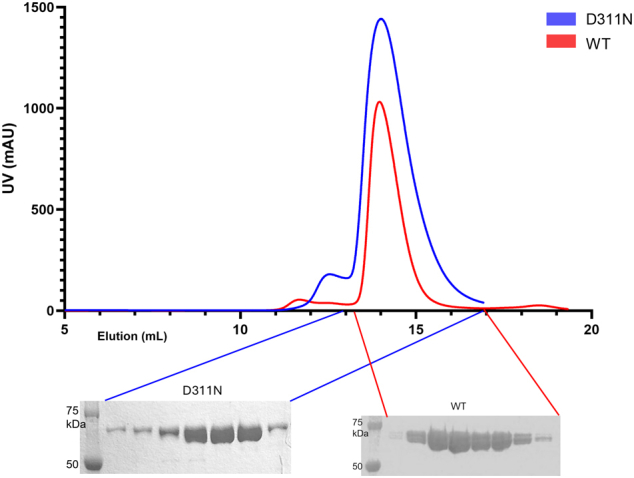
**SDS-PAGE gel of purified GRK5.** SDS-PAGE of GRK5_D311N_ elutions on a S200 size-exclusion chromatography compared to that of GRK5_WT_ which is heterogeneously phosphorylated and exhibits multiple bands.

The structures of *Escherichia coli*–expressed human GRK5_WT_ and GRK5_D311N_ in complex with Sgv were determined at resolutions of 2.8 and 2.6 Å, respectively, using 200 mM potassium citrate tribasic monohydrate (K_3_Cit·H_2_O) and 18 to 20% (w/v) polyethylene glycol 3350 (PEG3350) as a precipitant. Both structures were resolved starting from Met1 through residue Pro542. The linker between αN and α0 in the RH domain was not fully resolved (residues 19–20 in WT and 18–21 in D311N), and the extreme C terminus of the protein was disordered (residues 543–590) (Fig. 1, *A* and *B*). A global RMSD of 0.48 Å between the two structures for 538 Cα atoms indicates the two structures are very similar. The kinase domain conformation is most similar to that of GRK5 in complex with Ca^2+^·CaM and Sgv (Protein Data bank [PDB] entry: 6PJX), with a global RMSD of 1.31 Å for 541 Cα atoms *versus* the GRK5_WT_·Sgv structure. No density corresponding to the phosphate groups was observed in the autophosphorylated WT (Fig. S1) structure at either a known high abundance site (such as Thr10) or at heterogeneous sites in the kinase C-tail (Ser484 and Thr485) (19), suggesting that either these entities are too dynamic to observe or that nonphosphorylated species were selectively crystallized (Fig. 1*B*). The latter would seem to be true at least for the Thr10 site, which resides in a crystal lattice contact.

Sgv binds in the ATP-binding site of the kinase domain and replicates the interactions of adenine *via* hydrogen bonds with backbone atoms of hinge residues Thr264 and Met266. Its ribose group forms hydrogen bonds with the side chain of Asp270 and the backbone of Glu315. The AST, which is partially disordered in most GRK structures, is observed in both structures and stabilized by a network of interactions with αN, the small lobe, and the bound molecule of Sgv (Fig. 1, *C* and *D*). The phosphate binding loop (P-loop, residues 190–200) is also well resolved (Fig. 1*D*).

Unexpectedly, strong electron density consistent with a single heavy atom was observed in the large lobe at the turn between αD and αE. Because the atom responsible for this density is coordinated by the backbone carbonyls of residues His274, Met278, Asn280, and Gly282 and two water molecules, a water molecule is unlikely because it would have to donate at least four hydrogen bonds. By modeling various cations and considering the components of our buffer system, the density is most consistent with a K^+^ ion with distorted octahedral coordination and an average bond length of 2.8 Å (Figs. 1*B* and 4*C*) (20, 21). An analogous strong peak was also modeled as water at the same position in the previous GRK5·Ca^2+^·CaM structure, which could be due to the fact that the crystals were grown in potassium buffer (15). The next most likely cation would be Na^+^, but its average coordination distances are expected to be 2.4 Å, and when Na^+^ is modeled at this position, its *B*-factor is lower than surrounding atoms and it has positive 3.5 σ difference density, indicating that it has an insufficient number of electons to account for the data.

### Ligand-free structure of GRK5

**Figure 2:**
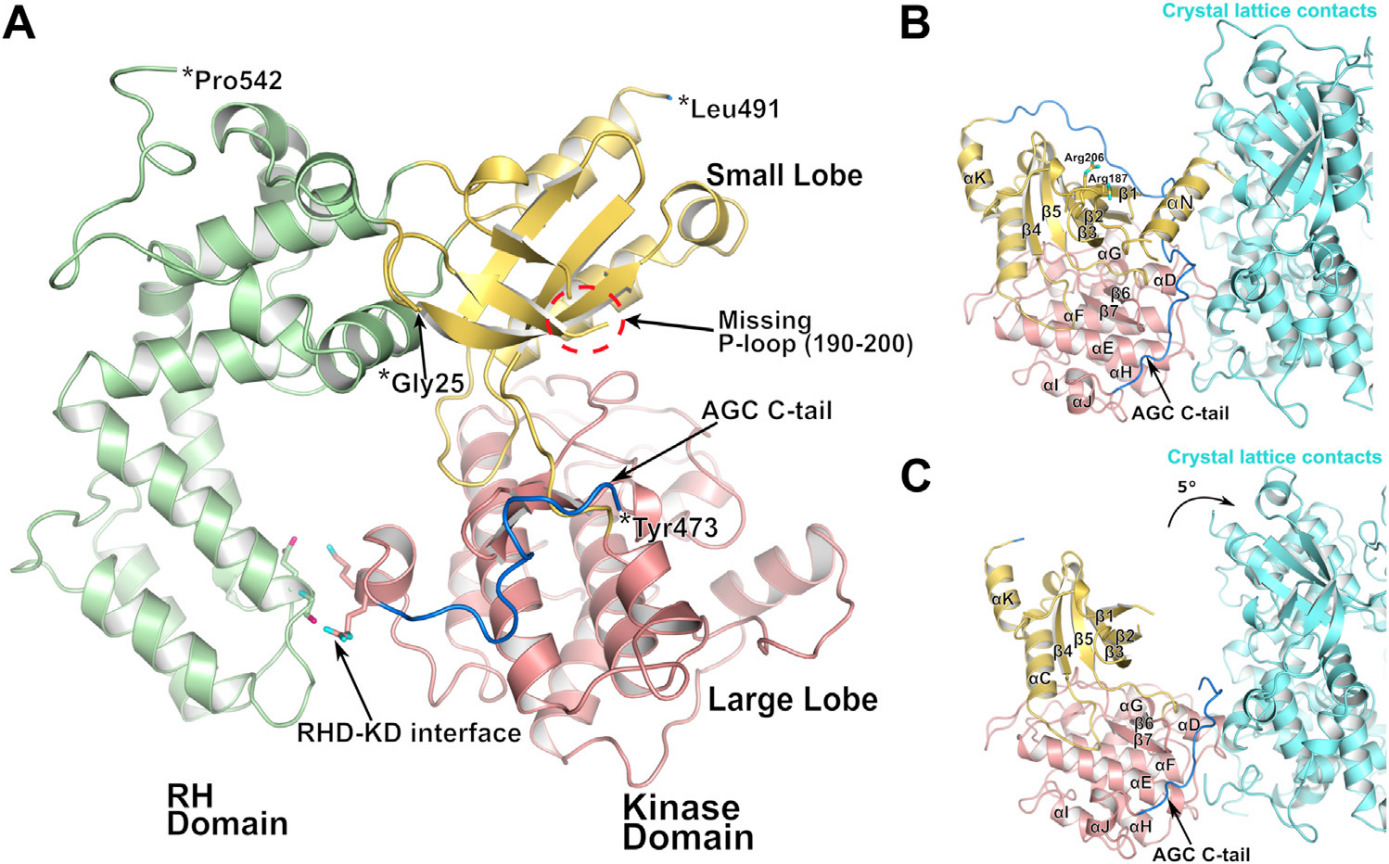
**Ligand-free structure of GRK5.***A*, crystal structure of ligand-free GRK5. Residues 1 to 24, 190 to 200 in the P-loop, and 474 to 490 in the AST are disordered relative to the Sgv-bound structure. In this state, a larger RHD–KD interface is formed. *B*, close-up view of Sgv-bound GRK5 lattice packing wherein a closed kinase domain is stabilized by αN in a crystal contact. *C*, close-up view of ligand-free GRK5. A ∼5° rotation of the lattice contact away from the small lobe occurs upon loss of K^+^. AST, active site tether; GRK5, G protein–coupled receptor kinase 5; αN, N-terminal α helix; RHD–KD, RH domain–kinase large lobe interface; Sgv, sangivamycin.

**Figure 3:**
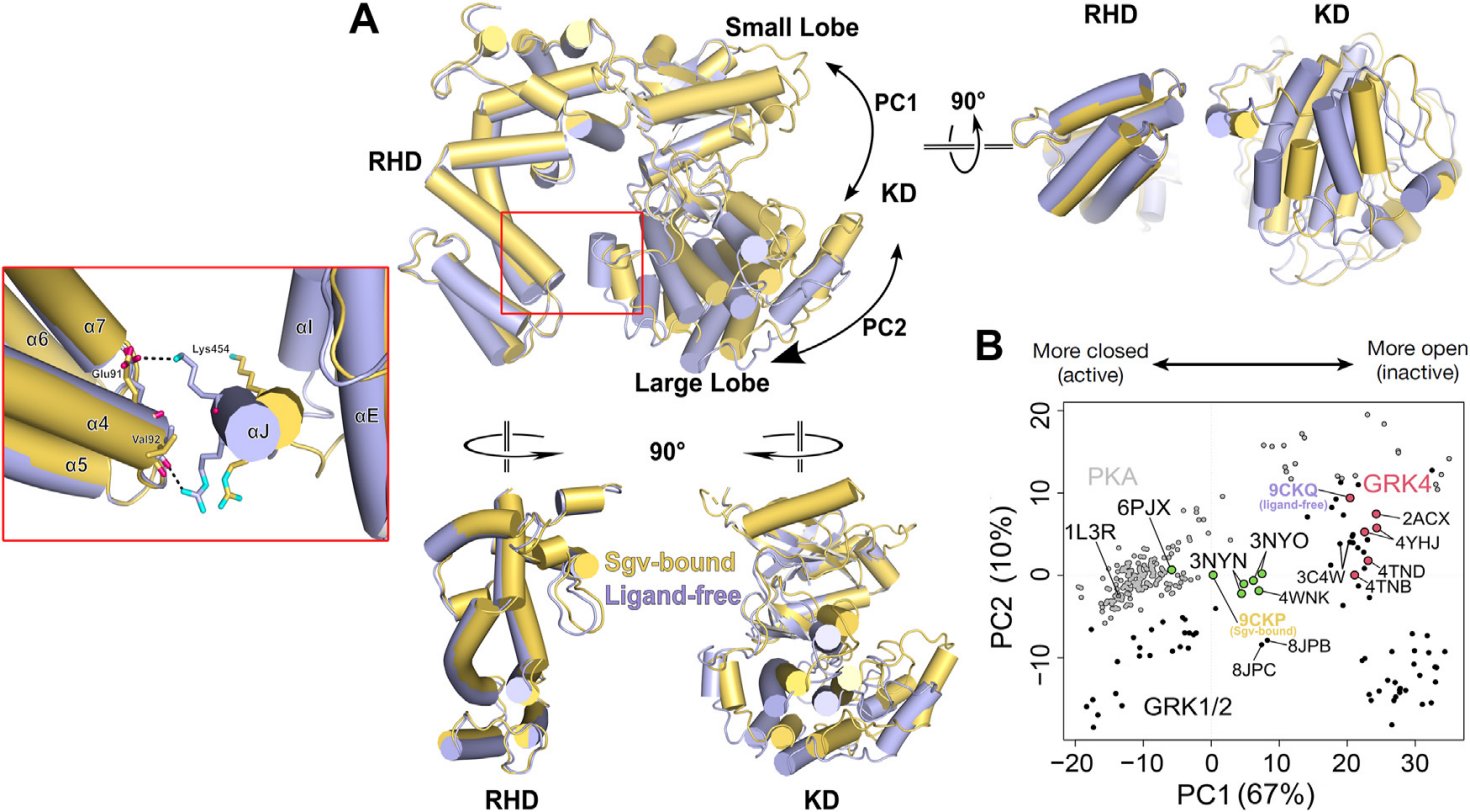
**Conformational comparisons of the kinase domains of GRKs and PKA.***A*, overlay of the Sgv-bound (*yellow*) and ligand-free GRK5 structure (*light purple*), aligning the small lobe (residues 180–270). The overlapped structures indicate minimal movement of the RH domain. The large lobe exhibits a ∼16° rotation away from the small lobe in the ligand-free structure. *Close-up inset* shows the RHD–KD interface between α4 and αJ was brought 3 Å closer in ligand-free structure, allowing a salt bridge to form between Glu91 and Lys454, and a hydrogen bond between Val92 and Arg455, along with increased van der Waals interactions. *B*, PCA plot analyzing PKA and GRK domains, with PC1 and PC2 corresponding to the two top transitions accommodating most variance (*A*). Each *circle* represents an experimental structure of a kinase domain. PKA structures are shown with *gray dots*, GRK1/2 subfamily structures with *black dots*, and GRK4 subfamily structures with *green* (more closed) and *red* (more open) *dots*. Two new structures presented in this article are labeled as “Sgv-bound” (PDB entry: 9KCP) and “ligand-free” (PDB entry: 9CKQ). The Ca^2+^·CaM–GRK5·Sgv structure (PDB entry: 6PJX) and the GRK6·Sgv–AMP structures (PDB entries: 3NYN and 3NYO) are indicated, as they represent the closest that GRK4 family members have previously approached the transition state-like structure of PKA (PDB entry: 1L3R). GRK, G protein–coupled receptor kinase; PCA, principal component analysis; PDB, Protein Data Bank; RHD-KD, RH domain–kinase large lobe interface; Sgv, sangivamycin.

**Figure 4:**
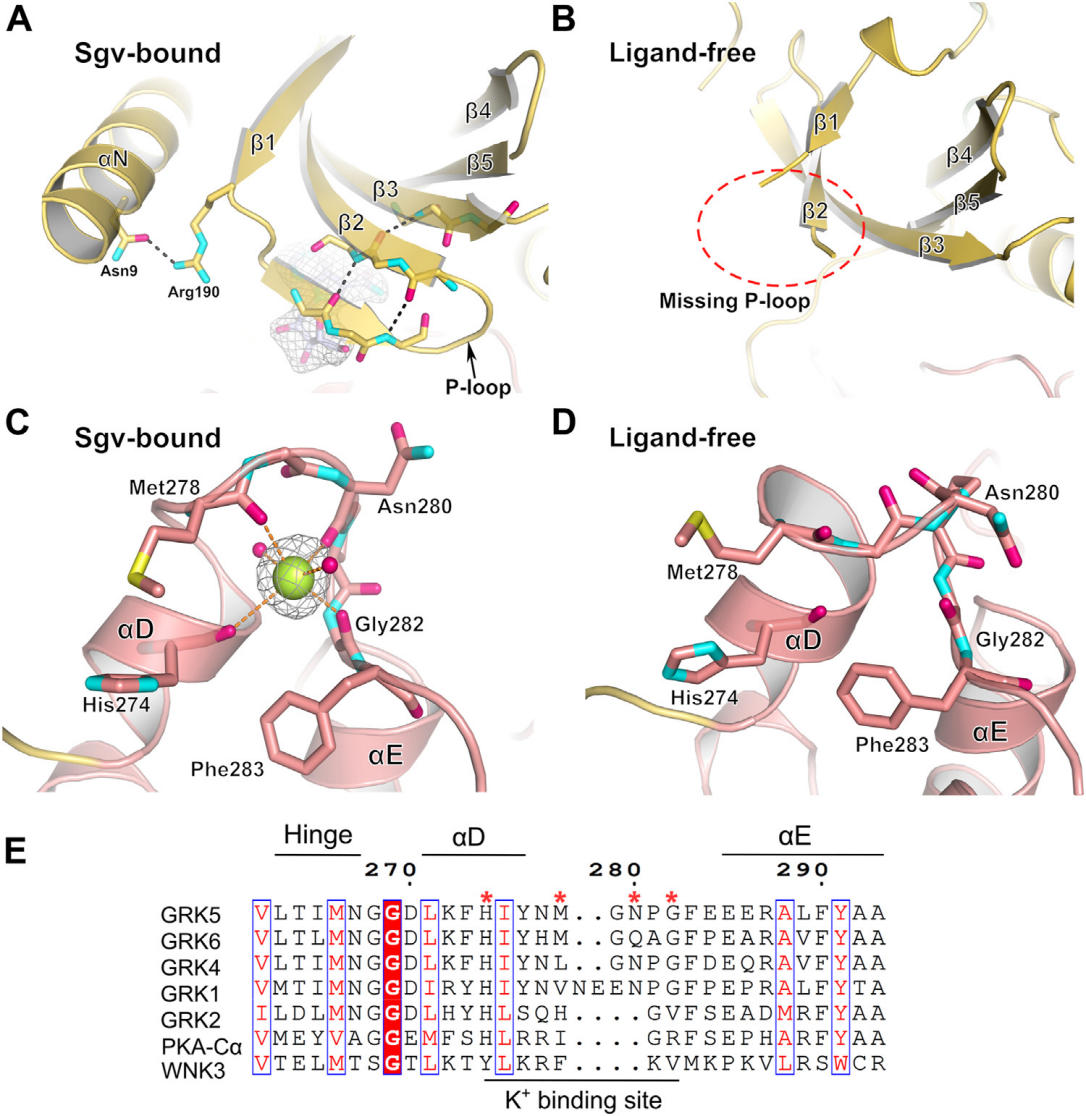
**Local conformational changes between Sgv-bound and ligand-free GRK5.***A* and *B*, in the Sgv-bound structure, stabilization of the P-loop results in extension of the β-sheet formed by β1, β2, and β3. *C*, a K^+^-binding site is formed by the αD–αE loop in the Sgv-bound structure, with K^+^ (*green sphere*) being coordinated (*orange dashes*) by the carbonyls of His274, Met278, Asn280, Gly282, and by two water molecules (*pink spheres*). Polder omit-map density for K^+^ is shown with a 5 σ cutoff. *D*, disruption of the site in the ligand-free structure because of extension of αD. *E*, sequence alignment of the GRK5 large lobe centered on the αD–αE region. *Red asterisks* denote residues with carbonyls that interact with K^+^. *Columns* with similarity score higher than 1 are considered as highly similar and are colored in *red* and framed in *blue*. Identical residues are highlighted in *red*. Multiple sequence alignment was generated with Clustal Omega (38, 39, 40) and rendered by ESPript 3.0 (41). GRK, G protein–coupled receptor kinase; Sgv, sangivamycin.

**Figure S2:**
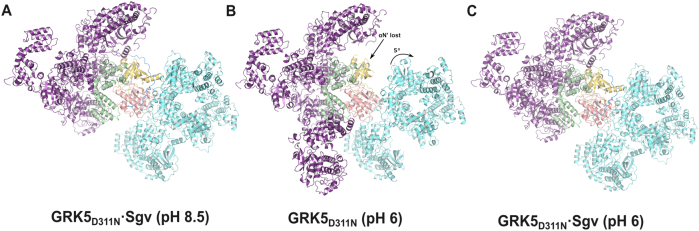
**Lattice packing in Sgv-bound and ligand-free GRK5 structures.** Crystal lattice contacts within 4 Å from the unique chain are shown. *A*, Lattice packing in the structure of the GRK5·Sgv complex. The unique chain has the same color scheme as in Figure 1. Purple symmetry mates pack against the RH domain, and cyan pack against the kinase domain. *B*, Lattice packing in the structure of ligand-free GRK5. An approximately 5° rotation centered from the large lobe away from the small lobe resulted in loss of density for αN’ and the AGC C-tail region. One additional lattice contact is formed in the ligand-free structure with the RH domain (lower symmetry mate). *C*, Low pH does not drive the conformational change from the structure in panels *A* to *B*. The lattice packing of GRK5·Sgv crystals soaked in 200 mM potassium citrate tribasic, 20% PEG3350 at pH 6 is the same as in in panel *A*.

The structure of ligand-free GRK5_D311N_ was determined at 2.7 Å using GRK5_D311N_·Sgv crystals transferred into a harvesting solution that omits Sgv and K_3_Cit but adds glycerol as a cryoprotectant. The crystal unit cell dimensions changed from 78 to 70 Å along the *c*-axis, with space group symmetry remaining the same (Table S1, Fig. S2). There is no density for Sgv or the K^+^ ion, and thus it is an entirely unliganded structure. The αN helix and the glycine-rich portion of the P-loop are disordered (Figs. 2*A* and 4, *A* and *B*) along with a segment of the AST from Cys474 to Asn490. In this crystal form, a lattice contact that stabilized the αN in the Sgv complex is rotated away from the small lobe by approximately 5° and no longer makes contact with GRK5 (Fig. 2, *B* and *C*). Aligning the small lobes of our ligand-free and Sgv-bound structures over residues 180 to 270 demonstrates a ∼16° difference in the angle between the large and small lobes (Fig. 3*A*). It has previously been noted that in the GRK4 family, there is a loss of contact between the RH domain and the large lobe (interface between α4 and αJ helices in each domain, respectively) accompanies its activation, driven by the motion of the large lobe away from the interface as the kinase domain closes (15). Indeed, the angle between α4 and αJ increased by 10°, and the distances between the residue pairs Glu91/Lys454 and Val92/Arg455 are 10 and 3 Å further apart, respectively, in the Sgv structure (Fig. 3*A*). These observations, along with what we now know from other “near active” structures of GRKs, are consistent with the need for an external hydrophobic surface, be it a GPCR, CaM, or crystal lattice contact, to stabilize the αN helix and allow it to pack with the kinase domain in a manner that promotes catalysis.

In this more open conformation, the K^+^ site is eliminated because the αD helix extends slightly and the backbone of Met278 encroaches on the metal site (Fig. 4, *C* and *D*). A sequence alignment of GRK5 with GRK1, GRK2, GRK4, GRK6, and PKA-Cα reveals high conservation in the αD and αE helices that bracket the K^+^-binding site. However, differences in packing of this region change the conformation of the backbone such that K^+^ is only compatible with the GRK5 Sgv-bound structure. “With No lysine (K)” kinases (WNKs), which regulate ion flux in the kidney, are also reported to be potassium regulated (22), but they are poorly conserved with GRK5 in the αD–αE region (Fig. 4*E*), and the configuration of their αD–αE loop is also incompatible with K^+^ binding, at least based on the available crystal structures, and thus their regulatory mechanisms likely differ.

### Conformational space of GRK4 subfamily structures

To better understand how these new structures relate to those previously reported for GRKs and PKA, principal component analysis was performed on the kinase domain of their deposited PDB entries. The two largest components of variance were found to be a “Pac-Man”-like opening and closing of the small and large lobes (PC1) and a more subtle twisting motion between the two domains (PC2) (23). The transition state complex of PKA (PDB entry: 1L3R; (24)) was used to define the expected “active conformation” of a GRK kinase domain, with the caveat that the GRK transition state configuration could be somewhat different.

Mapping our new Sgv-bound and ligand-free forms into this space places the new Sgv-bound structure between the PC1 coordinates of the Ca^2+^·CaM–bound GRK5 and Sgv-bound GRK6 structures, with the former being closer to the PKA transition state (Fig. 3*B*). The ligand-free GRK5 structure is located in the upper-right quadrant of the map, close to other GRK4-subfamily structures in inactive states. These include a structure of GRK4 (PDB entry: 4YHJ; (25)), GRK6 bound to AMP–PNP (PDB entry: 2ACX; (26)), and GRK5 bound to various ligands (PDB entries: 4TNB and 4TND (17); Fig. 3*B*). These are all found at a similar PC1 coordinate (22 Å) but vary over a 10 Å span along PC2, suggesting that the inactive state can accommodate a wide variety of interlobe twists, which is somewhat surprising because the open state features an additional contact between the RH domain bundle domain and the kinase large lobe (Fig. 3). Structures at smaller PC1 values (more closed) assume more similar PC2 values as they approach that of the transition state structure of PKA. Thus, the progression of GRK5 from an unliganded, open state to an “active-like” configuration primarily involves leftward migration along the PC1 axis (domain closure) and fixing the PC2 axis to a more restricted, common value.

### Structures of GRK5 bound to ATP·Mg^2+^–Mn^2+^

**Figure 5:**
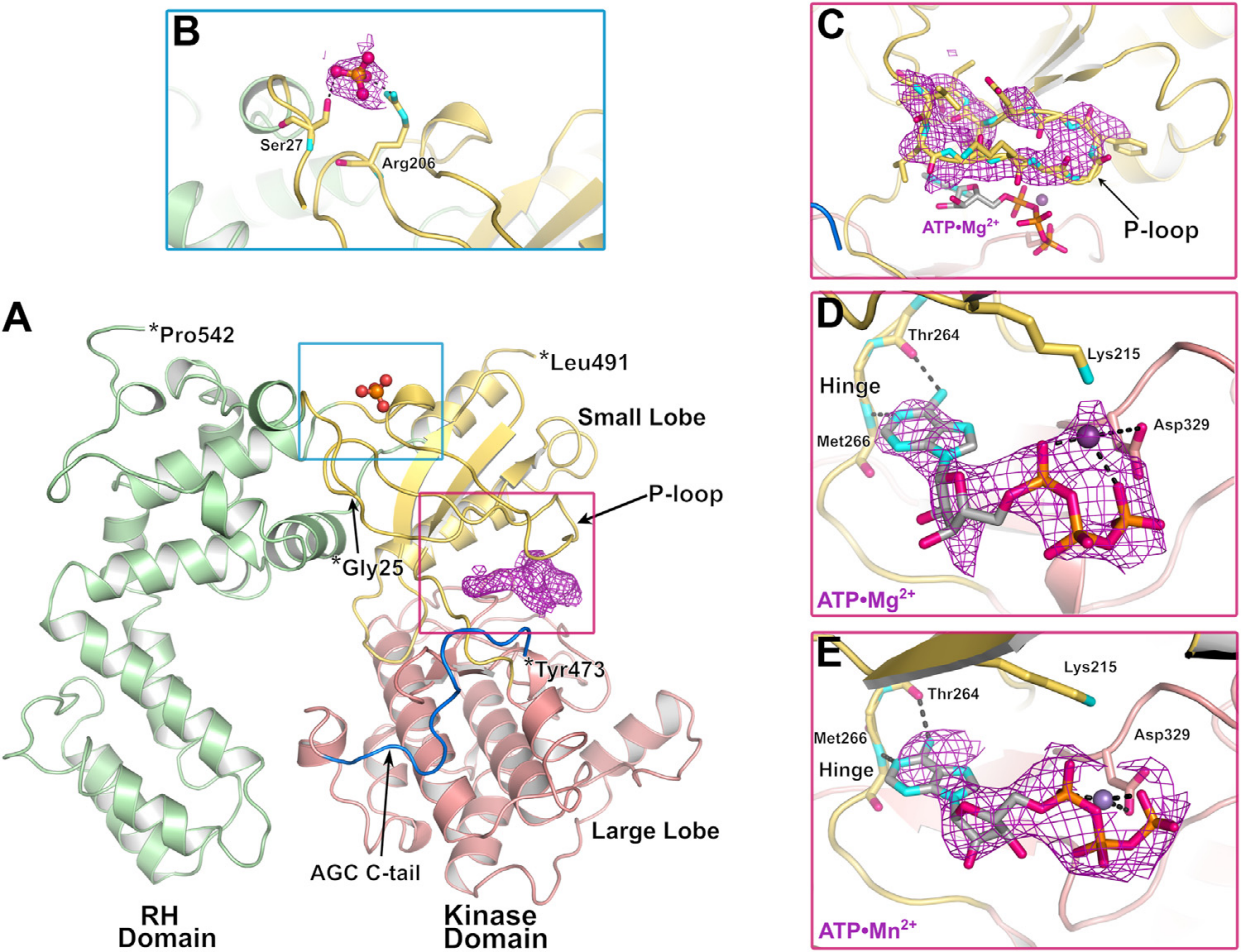
**Structures of GRK5 bound to Mg^2+^–Mn^2+^·ATP.***A*, the models are similar to ligand-free GRK5 and span residues Gly25 to Pro542 excluding a segment in AGC C-tail (residues 474–490). A phosphate, likely corresponding to a γ-phosphate of ATP included in the soak, is observed at a basic surface near the N terminus of the RH domain, where similar density was observed in the structure of the GRK6·AMP–PNP complex (PDB entry: 2ACX) (26). This could demark part of a binding site for the lipid phosphatidylinositol 4,5-bisphosphate, which positively regulates GRK5. *Red–violet mesh* is from a Polder omit map contoured at 2 σ. *B*, close-up view of the phosphate-binding site, where the anion makes hydrogen bonds with Ser27 and Arg206. *Red–violet mesh* corresponds to Polder omit map density contoured at 3.5 σ. *C*, Polder omit map density for the P-loop (residues 190–200) is shown at 2 σ, consistent with stabilization of the P-loop upon ATP binding. *D* and *E*, ATP (shown in *sticks*) binds to the active site along with 1 Mg^2+^ ion (*green sphere*) or Mn^2+^ ion (*purple sphere*). The metals are coordinated (*dashed lines*) by the α- and γ-phosphates and the side chain of Asp329. *Red violet mesh* is from a Polder omit map density contoured to 2.5 σ cutoff for ATP·Mg^2+^ and to 3 σ for ATP·Mn^2+^. AMP–PNP, adenylyl-imidodiphosphate; GRK5, G protein–coupled receptor kinase 5.

Although the Sgv-bound structure we report here has a near-active conformation, the same condition did not yield crystals when 350 μM Sgv was substituted by 1 mM ATP. Soaking ATP and metal ions directly into GRK5·Sgv crystals also failed to induce ligand exchange. We then soaked ATP·Mg^2+^ or ATP·Mn^2+^ using the same method used for inhibitors as described (18) with the hope of obtaining a unique ATP-bound state. After a 72-h soak, strong density was observed for both ATP·Mn^2+^ and ATP·Mg^2+^ in the active site and their crystal structures (resolutions of 2.8 and 3.0 Å, respectively). Similar to sunitinib-based inhibitor-bound structures of GRK5 (18, 27), the ATP-bound structures did not induce major changes compared with the ligand-free structure of GRK5, with a global RMSD of 0.35 Å for 490 Cα atoms (Fig. 5*A*). Relative to the unliganded structure, the P-loop becomes better stabilized in that there is stronger density for residues Lys194 to Glu199, which engage the ATP triphosphate (Fig. 5*C*). As expected, the adenine ring binds to the hinge with N1 forming a hydrogen bond with the backbone amide of Met266 and N6 binding to the backbone carbonyl of Thr264 (Fig. 5, *D* and *E*). We also attempted to reintroduce K_3_Cit back into the crystal after soaking with ATP in the open state to see if we could trap a more closed complex, but diffraction quality was severely compromised and no convincing conformational change in the kinase domain was observed.

**Figure S3:**
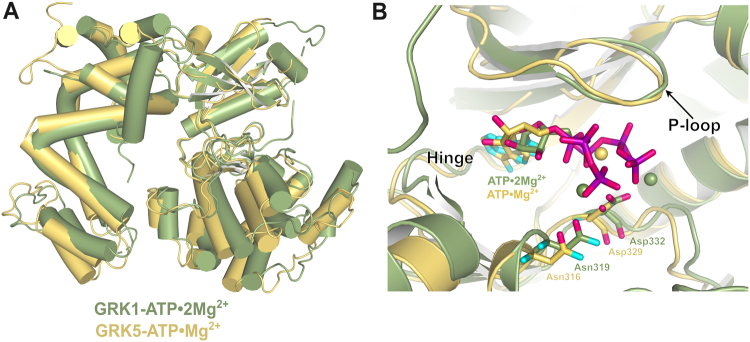
**Structural comparison between GRK5-ATP with GRK1-ATP.***A*, Overlay of GRK5 bound to ATP·Mg^2+^ (yellow) and GRK1 bound to ATP·2Mg^2+^ (3C4W, green), aligning the small lobe (residues 180 to 270). The large lobe exhibits a rotation away from the small lobe in GRK5 of ∼9° compared to that of GRK1. *B*, Close-up view of ATP binding site revealed that the phosphate/Mg^2+^-coordinating side chains (shown in sticks) are further away in the GRK5 structure.

In both structures, a divalent metal was modeled into the density and is coordinated by the α- and γ-phosphates and interacting with Asp329 (Fig. 5, *D* and *E*). Manganese ion is known to engage ATP with a higher affinity and enhance the binding of ATP and ADP to the active site, resulting in inhibition of kinase turnover rate (28, 29). The overall density for ATP·Mn^2+^ was indeed stronger compared with that for ATP·Mg^2+^ as indicated by a similar density at a 1 σ higher contour level (Fig. 5*E*). Compared with the structure of GRK1 bound to ATP·Mg^2+^ (PDB entry: 3C4W; (30)), the kinase domains of both GRK5·ATP structures are 9° more open resulting in a large difference in the orientation of the triphosphate group and its coordination with metal ions between the two homologs (Fig. S3). Nevertheless, the GRK1·ATP·Mg^2+^ structure is still not considered to be close to the closed, active conformation, given that it is located at a value of PC1 near other open, “inactive” structures (Fig. 3*B*).

### Removal of salt underlies the conformational change in GRK5

Crystallization of GRK5_D311N_·Sgv under our conditions occurred at pH 8.5, and transfer to a harvesting solution without K_3_Cit decreases pH to 6. To determine whether the structural transition was induced by lowering ionic strength or by the change of pH, the Sgv-bound crystals were soaked into a solution containing 200 mM K_3_Cit tribasic (pH 6), 20% PEG3350 for 5 min. The resulting space group and unit cell constants were similar to the closed form (Table S1, Fig. S2) and retained density corresponding to Sgv in the active site and K^+^ bound to αD–E. Thus, the change in conformation seems to be driven by loss of K_3_Cit, not pH.

### Both potassium and sodium salts inhibit GRK5 activity

**Figure S4:**
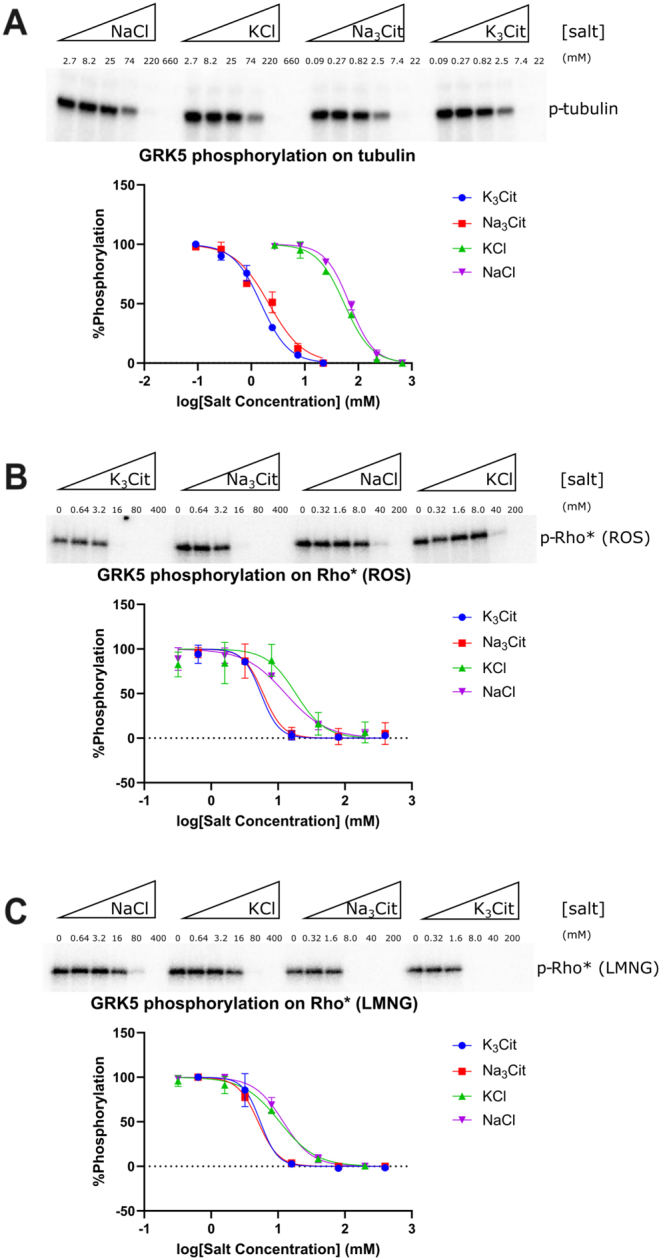
**Dose-dependent GRK5 inhibition in presence of K_3_Cit, Na_3_Cit, KCl, and NaCl.** The gel images shown are representative autoradiogram phosphorimages for ^32^P-labeled products *A,* tubulin; *B,* Rho∗ in ROS; ***C)*** Rho∗ in LMNG:CHS under a specific salt concentration. Dose curves were plotted with normalized data in each replicate, using the no-salt condition as 100% and blank as 0%. Three replicates from the same preparation of protein are plotted with error bars reflecting SD.

Because K^+^ is only observed in the two most “active” structures of GRK5 reported thus far, we wanted to test whether K^+^ can perturb kinase activation allosterically. It is well known that GRK activity is inhibited by NaCl (31), and in fact, most GRK assays are run at very low ionic strength for this reason. We speculated that K^+^ might selectively affect GRK5. Thus, we tested the effects of K_3_Cit tribasic, which was used at 220 mM to crystallize GRK5·Sgv, Na_3_Cit tribasic, KCl, and NaCl on the activity of GRK5 (Table 1). We used the soluble nonreceptor substrate tubulin and bovine light-activated rhodopsin (Rho^∗^) in both rod outer segments (ROSs) and in a micelle composed of lauryl maltose neopentyl glycol (LMNG) and cholesteryl hemisuccinate (CHS). Comparison of the activity with micelle-incorporated Rho^∗^ with that of Rho^∗^ in ROS tests if any differences in phosphotransfer are dependent on electrostatic interactions of GRK with the phospholipids. Comparison of tubulin phosphorylation with that of Rho^∗^ tests if ionic effects result from perturbation of ionic interactions with the receptor. With tubulin as the substrate, K_3_Cit and Na_3_Cit similarly inhibited GRK5 activity with IC_50_ values of 1.5 and 2 mM, respectively. Both KCl and NaCl inhibited GRK5 with about 35-fold lower potency than the corresponding citrate salts, far more than would be expected if one corrects for the effective concentration of either cation or anion (3-fold) or even the difference in ionic strength (6-fold). GRK2 and GRK6 exhibited very similar responses, indicating that the observed results were not dependent on any specific effects of K^+^ or other ions on GRK5. When using Rho^∗^ in ROS as the substrate, K_3_Cit and Na_3_Cit inhibited GRK5 activity at IC_50_ values of 6 mM, whereas the chloride salts inhibited at two to three-fold lower potencies, implying in this case, the difference could be due to the relative ionic strength when of K^+^ to citrate. The activity on Rho^∗^ solubilized in LMNG–CHS micelles gave similar results to ROS (Table 1, Fig. S4), implying that the inhibitory effects of the salts did not involve competition with the binding of charged lipid head groups to GRK5. Interestingly, the Hill slopes for inhibition of Rho^∗^ phosphorylation, whether in ROS or LMNG, were around −3.0. The basis for this apparent negative cooperativity is not known.

**Table 1:** Inhibition of GRK5 by various salts

Salt	GRK5 IC_50_ (μM)	GRK5 (Hill slope)	GRK2 IC_50_ (μM)	GRK2 (Hill slope)	GRK6 IC_50_ (μM)	GRK6 (Hill slope)	Substrate
K_3_Cit	1.5 ± 0.2 (3)	−1.6	0.8 ± 0.5 (3)	−1.3	0.9 ± 0.5 (3)	−0.9	Tubulin
Na_3_Cit	2.1 ± 0.5 (3)	−1.3	0.7 ± 0.2 (3)	−1.6	1.3 ± 0.9 (3)	−1.2	
KCl	55 ± 6 (3)	−1.7	20 ± 2 (3)	−1.6	14 ± 4 (3)	−2.3	
NaCl	69 ± 6 (3)	−1.9	21 ± 2 (3)	−1.2	8 ± 7 (3)	−1.3	
K_3_Cit	6 ± 2 (3)	−3.2	Not determined				Rho∗ (ROS)
Na_3_Cit	6 ± 3 (3)	−2.9					
KCl	18 ± 10 (3)	−1.9					
NaCl	13 ± 4 (3)	−1.4					
K_3_Cit	6 ± 2 (3)	−3.3					Rho∗ (LMNG)
Na_3_Cit	5.0 ± 0.5 (3)	−2.8					
KCl	11 ± 2 (3)	−1.6					
NaCl	12 ± 1 (3)	−2.0					

IC_50_ values for GRK5, GRK2, and GRK6 phosphorylation of the indicated substrates, shown as mean ± standard deviation calculated using four-parameter dose–response curves from three replicates. Example of dose–response curves is shown in Figure S4. Number of repeat measurements are shown in *parentheses*.

### Potassium ions selectively stabilize GRK5 in a closed state

**Figure 6:**
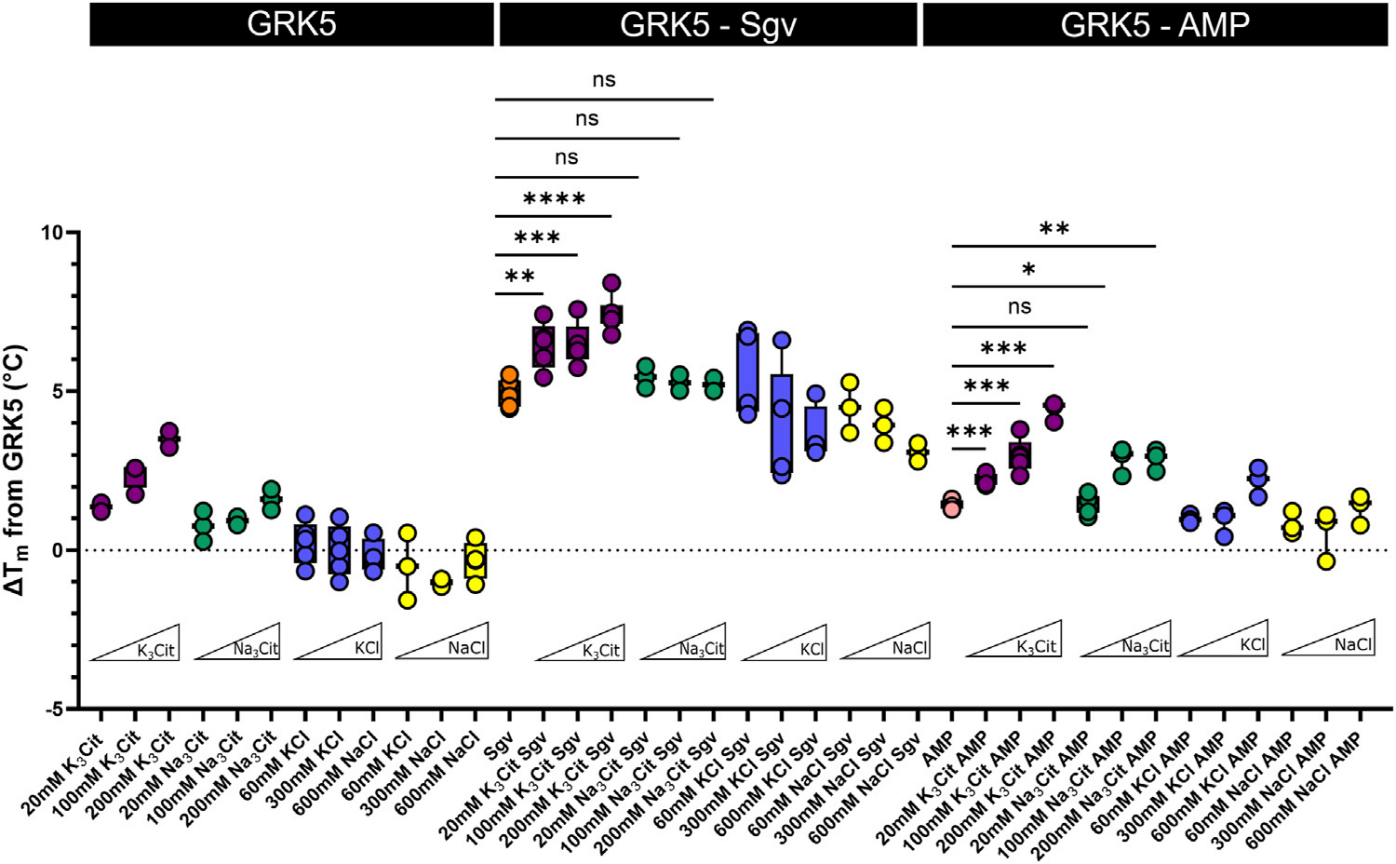
**Changes in GRK5 *T*_m_ upon addition of active site ligands and/or salt.** GRK5_WT_ at 2 μM was mixed with or without ligand (Sgv, AMP) in buffer containing 20 mM Hepes (pH 7.2), 2 mM MgCl_2_, in addition to a range of salt (K_3_Cit, Na_3_Cit, KCl, and NaCl) concentrations. Samples were mixed with Protein Thermal Shift dye to measure unfolding as a function of temperature. The *T*_m_ values were subtracted by the *T*_m_ of GRK5 in the same condition without the addition of salt to obtain Δ*T*_*m*_. Error bars correspond to SD (*box*) from three or more replicates (*circles*). Data are color-coded by the type of salt with K_3_Cit in *purple*, Na_3_Cit in *green*, KCl in *blue*, and NaCl in *yellow*. *Orange* corresponds to GRK5 with Sgv and *pink* with AMP in buffer alone (20 mM Hepes [pH 7.2], 2 mM MgCl_2_). A one-tailed *t* test was used to determine significance (ns: *p* > 0.05, ∗*p* ≤ 0.05, ∗∗*p* ≤ 0.01, ∗∗∗*p* ≤ 0.001, and ∗∗∗∗*p* ≤ 0.0001). GRK, G protein–coupled receptor kinase; ns, not significant; Sgv, sangivamycin.

Although we found no evidence that K^+^ is activating GRK5, our structural studies suggested that potassium salts should still stabilize GRK5 more than sodium salts, but only in the presence of ligands that stabilize a more closed state, such as Sgv. To test this, we measured the *T*_m_ of GRK5 at salt concentrations close to their IC_50_ values (Table 1, Fig. S4) as well as at the ionic strength used for crystallization (Fig. 6). Addition of K_3_Cit at increasing concentrations raised the *T*_m_ of GRK5 proportionally. Na_3_Cit exhibited a similar but weaker effect, suggesting that citrate has a stabilizing effect on its own, and that the potassium salt stabilizes at least more than Na^+^. KCl did not increase GRK5 stability at any concentration, whereas NaCl reduced it. Thus, chloride salts are thus destabilizing compared with their citrate counterparts, but the presence of K^+^ still increased stabilization relative to Na^+^. Sgv increased the *T*_m_ by 5 °C relative to unliganded GRK5, which was further increased by K_3_Cit but not by Na_3_Cit. This implies that citrate ions do not offer net stabilization to Sgv-bound GRK5. Thus, K^+^ can significantly stabilize the Sgv-bound state, but only when the counterion is not chloride. AMP, which could substitute for Sgv in crystallization trials of GRK6 (12), elicited analogous results but at a lower *T*_m_ because AMP itself does not stabilize GRK5 as much as Sgv (Fig. 6).

To examine whether K^+^ had an effect on the binding of physiological ligands to GRK5, we measured the *T*_m_ in the presence of the substrate ATP and the product ADP (Fig. S6*A*). ATP·Mg^2+^ binding to GRK5 increased the *T*_m_ by 9 °C, which was decreased upon addition of increasing concentrations of KCl and NaCl. Surprisingly, at 20 mM K_3_Cit, the *T*_m_ decreased by 2 to 3 °C, but this was gradually restored by increasing the concentration to 200 mM. Na_3_Cit did not cause any significant changes in *T*_m_. Repeating the *T*_m_ measurements for K_3_Cit between 0 and 220 mM with additional data points taken between 0 and 20 mM, biphasic behavior was observed: a dose-dependent decrease in the *T*_m_ from 0 to 20 mM, followed by an increase (Fig. S6*B*). Binding of K^+^ may be responsible for the initial decrease in stabilization if it is favoring a conformation that is not compatible with ATP, as it would also be consistent with the IC_50_ value we measured for K_3_Cit against Rho^∗^ (Table S2). The fact that Na_3_Cit does not induce the same dip in stability supports this conclusion. Citrate thus can restore GRK5 to the same level as the ATP-stabilized configuration, which suggests it might be binding in the active site in competition with triphosphate.

**Figure S5:**
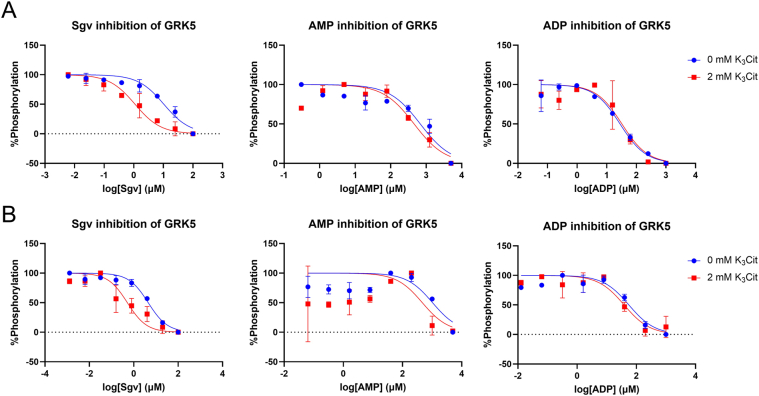
**Inhibition of GRK5 by adenosine analogs in the presence or absence of K_3_Cit.** Dose-dependence of GRK5 activity towards *A*, tubulin or *B,* Rho∗ in presence of ligands (Sgv, AMP, ADP) with or without K_3_Cit. Two replicates from separate experiments using the same preparation of protein were plotted for mean and standard deviation of IC_50_ values.

If K^+^ selectively stabilizes the binding of Sgv to GRK5, then it should augment inhibition of GRK5 by Sgv. To test this, we measured the ability of 2 mM K_3_Cit (close to its IC_50_ value) to enhance the inhibition by Sgv, AMP, and ADP of GRK5 phosphorylation of tubulin and Rho^∗^ (Table S2, Fig. S5). Only the Sgv curve was left-shifted, reducing the apparent IC_50_ for Sgv eightfold, indicating that Sgv and K_3_Cit inhibit GRK5 synergistically. Sgv inhibition of Rho^∗^ phosphorylation was also shifted, with the IC_50_ values reduced sevenfold. K_3_Cit, however, did not cause changes in either AMP or ADP inhibition potency.

To test whether the effects of K^+^ were specific for GRK5, we tested the same conditions on GRK6, a close homolog, and GRK2, representing another GRK subfamily. For GRK6, all salts exhibited destabilization whether in the presence or the absence of Sgv (Fig. S7), and for GRK2, similar patterns of destabilization were observed under each condition (Fig. S8), regardless of their differences in basal *T*_m_ and in Δ*T*_*m*_ upon ligand binding (Fig. S9). Overall, the results are consistent with the K^+^-binding site being unique to GRK5.

## Discussion

The six atomic structures of GRK5 reported herein represent two distinct states of GRK5. The first is compatible with Sgv and is relatively closed and most similar to the conformation of transition-state PKA (Fig. 1). The second state is formed upon removal of K_3_Cit, wherein the structure relaxes into a conformation compatible with the binding of small-molecule active-site inhibitors (18, 27), ATP·Mg^2+^, and ATP·Mn^2+^ (Figs. 2, 5). We showed that the conformational transition is not because of an associated drop in pH (Fig. S2*C*). There was also no convincing density for citrate ions in the GRK5·Sgv complex, but there was for K^+^, which binds at a site close to the kinase domain hinge that is eliminated upon transition to the open state. Thus, loss of K^+^ is most likely responsible for the remarkable structural transition we see in these GRK5 crystals.

**Figure 7:**
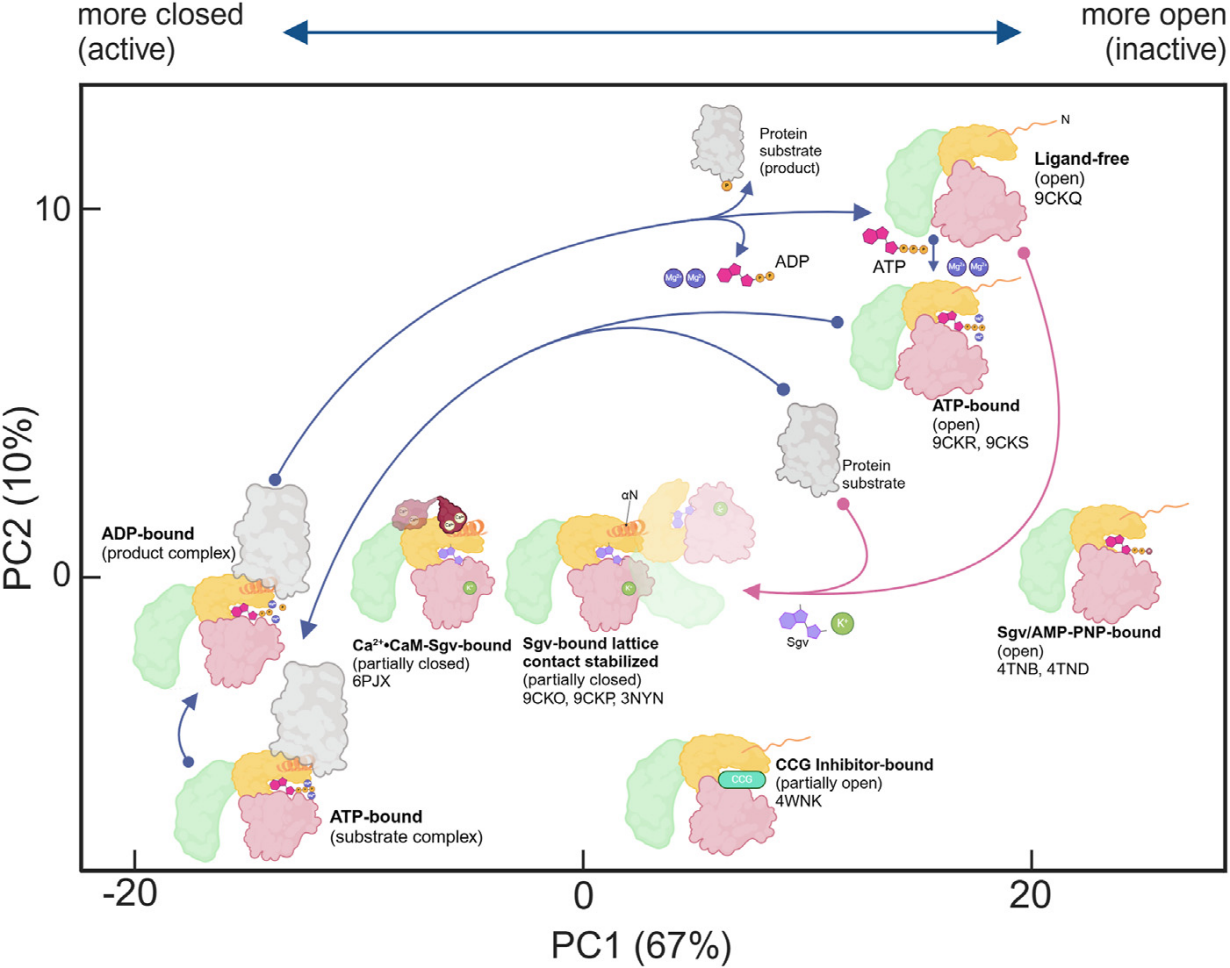
**The GRK5 catalytic cycle mapped in PC space.** Ligand-free GRK5 exhibits an open conformation in the upper-right corner of the PCA plot (*cf.*Fig. 3*B*). ATP and Mg^2+^ ions can bind into the active site in this conformation, and then binding of a protein (CaM or GPCR) induces formation of αN (*orange spiral* when helix formed and *squiggle line* when disordered) and drives closure of the kinase domain. Release of ADP occurs upon opening of the kinase domain, although it is not known if this involves complete dissociation from the target protein. On the other hand, the binding of Sgv and a K^+^ ion promotes kinase domain closure to a distinct partially closed conformation. As indicated by the 4WNK structure (shifted downward in PC2 for clarity) (16), this partially closed state does not also feature an ordered αN if there is not an appropriate protein–protein interface formed by the N terminus of αN. PC coordinates of the closed ATP- and ADP-bound states are hypothetical and positioned based on PKA structures in the same sates. GRK5 small lobe is shown in *yellow*, large lobe in *pink*, and RH domain in *light green*. Created in BioRender. Chen, Y. (2024) https://BioRender.com/o35n760. CaM, calmodulin; GPCR, G protein–coupled receptor; GRK5, G protein–coupled receptor kinase 5; αN, N-terminal α helix; PCA, principal component analysis; Sgv, sangivamycin.

This raised the possibility that K^+^ could be a physiological regulator of GRK5. There is precedence for this idea because K^+^ is also a proposed regulator of WNK kinases (22). Because the Sgv-bound conformation is closer to the transition-state–like conformation of PKA (Figs. 3*B* and 7), we first speculated that the cation might be able to promote activity. Testing this idea is complicated by the fact that high ionic strength is well known to inhibit GRK activity and that GRKs are potently inhibited by polyanions such as heparin (32) and RNA aptamers (29). However, we could not discern any positive effect of K^+^ on the ability of GRK5 to phosphorylate either the soluble substrate tubulin, or Rho^∗^ whether in ROS or reconstituted in LMNG–CHS micelles, although there were some interesting observations, such as more potent inhibition of tubulin phosphorylation by citrate salts relative to chloride (10-fold or greater), an effect that was largely muted when considering Rho^∗^ phosphorylation (2-fold to 3-fold). Another observation was evidence for negative cooperativity (Hill slopes <−1) only in inhibition of Rho^∗^ phosphorylation by citrate salts. We could not detect a difference in GRK6 or GRK2 phosphorylation of tubulin as a function of K^+^. Consistent with this, examination of all the reported structures of GRK2 and GRK4 subfamily members suggests that the K^+^-binding site is found only in GRK5. The K^+^ site is also not formed in any reported WNK3 structure, and thus the mechanism of regulation for GRK5 and WNK3 by potassium ions is likely distinct. Despite being relatively closed in comparison (Fig. 3*B*), the 4WNK (PDB entry) structure of GRK5 also lacks the metal-binding site (16). Thus, it is only observed when αN is docked to the GRK5 hinge, such as in our GRK5–Sgv complexes and in the Ca^2+^·CaM–GRK5 complexes (15). The 4WNK structure also demonstrates that a closed kinase domain conformation such as those adopted by our GRK5–Sgv complex and the Ca^2+^·CaM–GRK5 complex is not sufficient to stabilize and bind the αN helix. There apparently needs to be an external interaction with the extreme GRK N terminus to stabilize the αN helix, an interaction that is lacking in the 4WNK structure.

**Figure S6:**
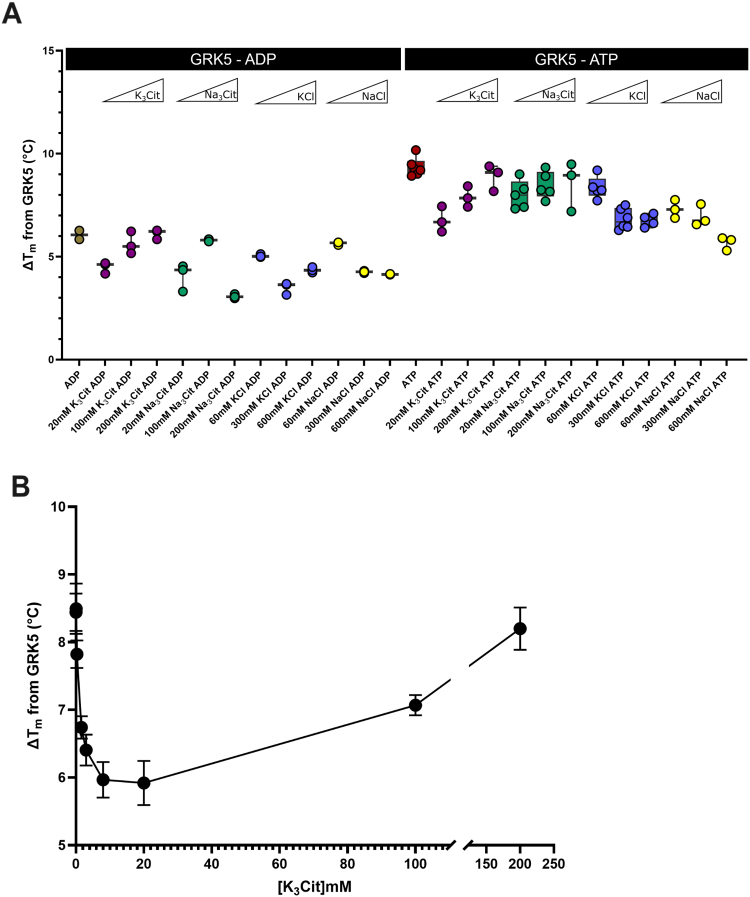
**Changes in T_m_ of GRK5 upon addition of ADP/ATP and various salts**. T_m_ values of GRK5_WT_ were measured under the same conditions as mentioned in Figure 6 with ligands ADP/ATP. *A*, Three or more replicates from separate experiments (circle) are shown along with their SD (box). *B,* Dose-dependent change in ΔT_m_ of GRK5 in the presence of ATP versus increasing concentrations of K_3_Cit appeared biphasic. This behavior is not replicated by Na_3_Cit suggesting that K^+^ is stabilizing a distinct conformation less able to bind ATP.

**Figure S7:**
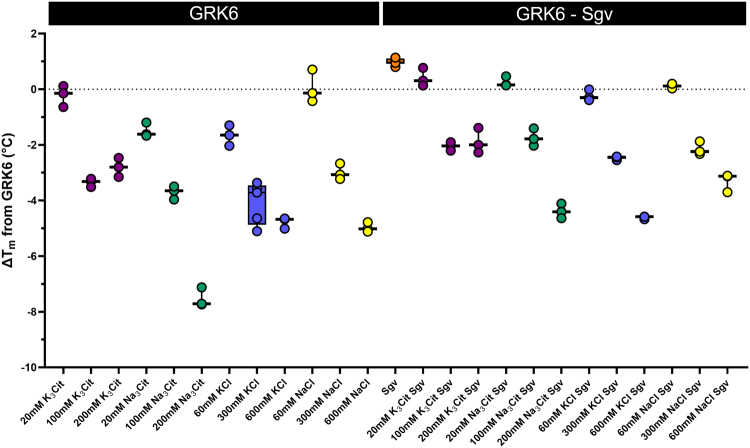
**Changes in T_m_ of GRK6 upon addition of Sgv ± salt**. T_m_ of GRK6 measured under the same conditions as in Figure 6. Three or more replicates from separate experiments (circles) are shown along with their SD (box). Note that differences in the Sgv-induced shift in T_m_ for GRK6 compared to GRK5 may partially reflect different basal T_m_ values in that both GRK2 and GRK6 have a higher T_m_ than GRK5 under basal conditions (Figure S9).

**Figure S8:**
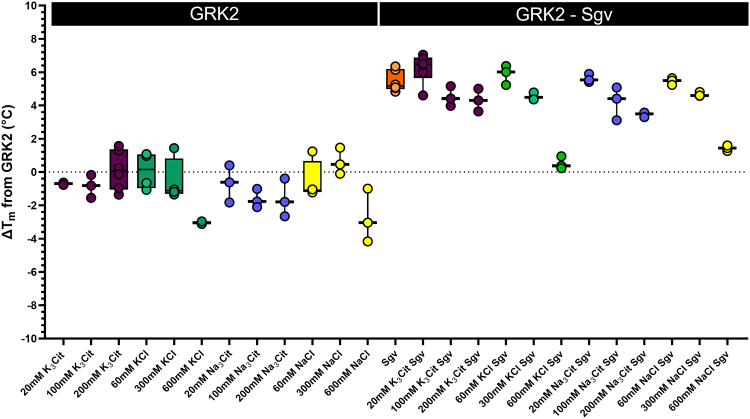
**Differences in T_M_ of GRK2 upon addition of ligands ± salt**. T_m_ of GRK2 measured in the same conditions as in Figure 6. Three or more replicates from separate experiments (circles) are shown along with their SD (box). Significance of GRK2 with Sgv alone were compared to those with added K_3_Cit using a one-tailed t-test, and the resulting *p* values shown in corresponding labels (ns: *p* > 0.05, ∗: *p* ≤ 0.05). Note that differences in the Sgv-induced shift in T_m_ for GRK2 compared to GRK5 may partially reflect their intrinsic basal T_m_ values. Both GRK2 and GRK6 have a higher T_m_ than GRK5 under these conditions (Figure S9).

**Figure S9:**
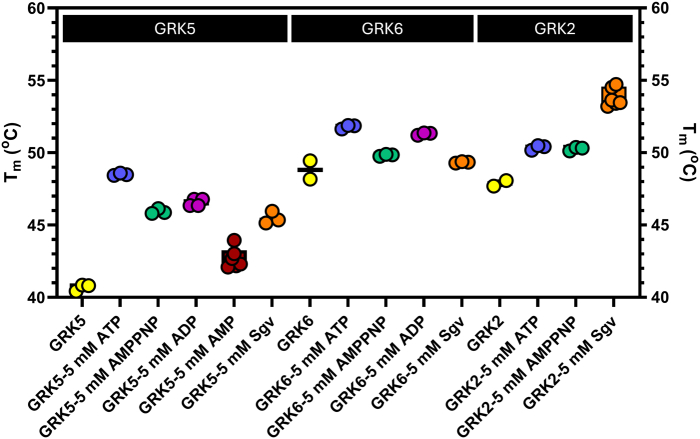
**T_m_ of GRK2, GRK5, and GRK6 ± ligands**. T_m_ is shown as the mean ± SD (box) from three separate experiments shown in individual point (circle) using the same preparation of protein. Bars are color-coded as GRK-only in yellow, with ATP in blue, AMPPNP in green, ADP in purple, and Sgv in orange.

Based on these data, we propose that K^+^ can trap GRK5 in a relatively closed, dead-end complex incompatible with ATP. This idea is supported by the facts that K_3_Cit significantly increases the stability of GRK5 alone or in complex with either Sgv or AMP, that K_3_Cit increases the potency of Sgv inhibition of tubulin and receptor Rho∗ phosphorylation by ∼8-fold (Table S2, Fig. S5), and that K_3_Cit decreases the stability of the more physiological GRK5–ATP complex (Fig. S6*A*). Our analysis of physiological ligands however showed mixed behavior, with generally no significant effect on stabilization of ADP binding and more complex effects on ATP binding (Fig. S6). For GRK2, all salts instead tended to decrease stability as a function of increasing concentration (Fig. S8). Despite a highly similar conformation in the Sgv-bound GRK6 structure (PDB entry: 3NYN; (12); RMSD of 2.64 Å for 532 Cα), GRK6 was also destabilized by all additions of salt (Fig. S7).

GPCRs can regulate G protein–coupled inwardly rectifying potassium channels (GIRKs) *via* release of free G_βγ_ subunits (33, 34), which leads to membrane depolarization. Previously, it was found that GRK2/3 can inhibit GIRK currents by competing for G_βγ_ (35). The K^+^ concentration is ∼140 mM inside polarized cells, a concentration that would allow for full inhibition of GRK5. Therefore, the potassium-binding site in GRK5 may also play a role in desensitizing GPCRs in this process by reduced tonic K^+^ inhibition. Upon depolarization, the internal concentration of K^+^ does not change much overall, but in the vicinity of active channels, it could be quite low, leading perhaps to higher GRK5 activity and more rapid local GPCR desensitization. Physiologically, this would make sense because GIRKs are primarily found in the heart and central nervous system where GRK5 is abundant. Further studies are required to test this idea.

There is as of yet no reported structure of a GRK bound to either ATP or an ATP analog in which the kinase domain assumes a closed, catalytically competent configuration. Here, we were also unable to generate a closed conformation of GRK5 when substituting ATP for Sgv during crystallization, and structures of the unliganded, open state of GRK5 soaked with ATP·Mg^2+^–Mn^2+^ only exhibited stabilization of the glycine-rich P-loop. These open structures likely represent a “loading” or “product release” state in which the catalytic residues are not in the correct configuration to drive phosphotransfer, but these ligands can freely enter or leave the active site. In contrast, the kinase domain of PKA can close and catalyze phosphotransfer simply upon binding ATP. This makes sense because the key regulatory step for PKA is the binding of cAMP to and release of its regulatory subunits, and the kinase has a strong target consensus sequence that determines which proteins will be phosphorylated. A failure to close upon binding ATP serves to prevent phosphotransfer by GRK5 until an active GPCR (or Ca^2+^·CaM) docks to the GRK (Fig. 3*B*). This is especially important because phosphotransfer in GRKs is relatively indiscriminate in that they do not have a strong target consensus sequence, and that individual GRKs must be able to phosphorylate hundreds of GPCRs that have highly diverse intracellular loops and tails.

In summary, the new structures reported here have expanded our understanding of the conformational transitions available to the GRK4 subfamily of GRKs. Referring to PC space (Fig. 3*B*), the Sgv-bound structure we report is relatively close to the CaM-bound structure but slightly more positive on PC1 axis, and the ligand-free structures of GRK5 we report occupy the upper right quadrant of the plot, with the highest PC2 coordinates but a similar PC1 coordinate to other previously reported GRK4-subfamily structures. In the open state, there is clearly plenty of conformational flexibility in the PC2 (wag) dimension, and a tightening of PC2 as structures progress toward the transition state PC1 value, similar to those observed for GRK1 in complex with Rho^∗^ (13). It is not surprising, given the fact they are both members of the AGC kinase subfamily, that the various PKA structures follow a similar transitional path from open to closed. Because neither GRK6·Sgv crystals (12) nor our GRK5·Sgv crystals could be reproduced with either ADP or ATP, and because K^+^ can stabilize the Sgv-bound but not the ATP-bound form of GRK5, we are forced to conclude that the Sgv-bound states of GRK5 and GRK6 are not “active states.” A possible explanation that the P-loop glycine-rich segment in both the Sgv-bound structures encroaches into the triphosphate-binding site, rendering it incompatible with ADP or ATP. We speculate based on these structures that the formation of a bipartite complex with tan activated GPCR (*e.g.*, the C terminus of a GPCR in addition to its transmembrane core) may be the required final step to stabilize a closed, ATP-bound state. In the future, structures of GRKs in ATP–ADP-bound, closed states are therefore a high priority.

## Experimental procedures

### Protein production

Following the protocol in Ref. (19), pMAL-vector plasmids bearing DNA encoding human GRK5_WT_ or GRK5_D311N_ (residues 1–590 followed by a hexahistidine tag) were transfected into *E. coli* Rosetta (DE3) and induced with isopropyl β-d-1-thiogalactopyranoside. Cells lysed using an Emulsiflex-C3 were treated with DNase I and 1 mM MgCl_2_ at room temperature for 10 min before centrifugation at 40,000*g*. GRK5 in the soluble fraction was then loaded onto a Ni^2+^–NTA column for metal-affinity purification. Eluted GRK5 fractions were then loaded onto a tandem HiTrap Q HP-HiTrap SP HP column to capture impurities in the Q HP column, and GRK5 was eluted from the SP column using an NaCl concentration gradient from 100 to 600 mM. Finally, GRK5 fractions were pooled, concentrated, and loaded onto a Superdex 200 Increase 10/300 GL gel filtration column to obtain >95% purity of GRK5 in a buffer containing 20 mM Hepes (pH 7.2), 100 mM NaCl, 0.5 mM Tris(2-carboxyethyl)phosphine) (TCEP). Purified GRK5 was used directly in crystallization or flash-frozen with liquid nitrogen and stored at −80 °C for later usage. Human GRK2_S670A_ and GRK6FL_pal(−) are expressed in Sf9 insect cells and purified as described previously (26, 31). The S670A mutation in GRK2 eliminates a heterogenous mitogen-activated kinase phosphorylation site, and GRK6FL_pal(−) was used to remove palmitoylation sites in order to make expression and purification easier.

### Protein crystallization, ligand soaking, and harvesting

GRK5 in complex with Sgv was crystallized at a final concentration of 118 μM GRK5, 354 μM Sgv, and 118 μM MgCl_2_. Crystals were obtained by mixing 2 μl of protein (in buffer containing 20 mM Hepes [pH 7.2], 100 mM NaCl, and 0.5 mM TCEP) with 2 μl well solution composed of 220 mM potassium citrate tribasic and PEG3350 (20–25% for WT and 18–20% for D311N) and suspended in hanging drop format over 600 μl well solution at 4 °C for 1 week. Single crystals were frozen in LN_2_ using 10% glycerol as a cryoprotectant in addition to the PEG3350 in the well solution. For the ligand-free structure, the crystals were transferred to a soaking condition containing 20 to 25% PEG3350 and 10% glycerol for at least 1 min before freezing. For ligand-bound structures, the soaking condition was supplemented by either 1 mM ATP and 1 mM MgCl_2_ or 500 μM MnCl_2_ on a coverslip in a sealed hanging drop apparatus with 20% PEG3350 and 10% glycerol in the well for 3 days before freezing, similar to inhibitor soaking as mentioned (18, 36). All crystal soaking and harvesting procedures were performed at 4 °C.

### Data processing and structure refinements

Diffraction data were collected at the Advanced Photon Source (APS) at a wavelength of 1.0332 Å or at National Synchrotron Light Source II at a wavelength of 0.9201 Å. Data were collected in 1° wedges per frame for a total of 180 frames. Automated-processed data from DIALS *via* Xia2 (APS) or from Fast Data Processing was used, and phasing was accomplished *via* molecular replacement in PHENIX, with PDB entry 6PJX as the search model for Sgv-bound structures, or with a GRK5 model based on PDB entry 2ACX for the ligand-free structure or complexes with ATP. Refinements were performed using PHENIX alternating with manual building and fitting in Coot (crystallographic object-oriented toolkit). The final models were validated with MolProbity prior to deposition along with structure factors in the PDB. Atomic figures were created with PyMOL 3.0 by Schrödinger (37). Multiple sequence alignment was generated with Clustal Omega by EMBL-EBI (38, 39, 40) and rendered by ESPript 3.0 for visualization (41).

### Kinase inhibition assay

GRK inhibition assays were performed in 20 mM Hepes (pH 7.0), 2 mM MgCl_2_, 0.025% n-dodecyl-β-d-maltoside with 50 nM human GRK5_WT_ as purified previously. GRK5 was mixed with 500 nM porcine brain tubulin (PurSolutions) or Rho (inactive rhodopsin) purified from ROS or Rho solubilized in 0.5% LMNG–0.05% CHS micelles at the final concentration of 50 nM of GRK5 and 500 nM of tubulin or Rho. A range of salt concentrations was added 30 s prior to 5 min reactions initiated by exposing samples to light followed by addition of 5 μM ATP supplemented with radioactive [γ-^32^P]-ATP at 60 Ci/mmol (PerkinElmer Life Sciences) at room temperature. To test the effects of K_3_Cit in ligand inhibition, a range of concentration of ligands (Sgv, ADP, and AMP) were added to the reaction 30 s prior to reaction initiation with 2 mM of K_3_Cit. Reactions were quenched with 4X SDS loading buffer, separated in SDS-PAGE, dried, and read by phosphorimaging and then quantified *via* a Personal Molecular Imager and Quantity One 1-D Analysis Software. Data were analyzed *via* GraphPad Prism (GraphPad Software, Inc), and three-parameter dose-dependent curves (Hill coefficient = −1) plotting phosphate transferred against inhibitor concentration were used for the calculation of IC_50_ values. At least three replicates were obtained to calculate the mean and standard deviation of IC_50_ values. See Figure S4 for representative raw and processed data.

### Differential scanning fluorimetry thermal shift assay

Full-length WT GRK5, GRK2, or GRK6 at a final concentration of 2 μM in the absence or the presence of different ligands were mixed with the Protein Thermal Shift Dye at a final concentration of 1X in buffer containing 20 mM Hepes (pH 7.2), 2 mM MgCl_2_, and 1 mM TCEP in addition to different concentrations of salts. The melting points were measured using a QuantStudio5 Real-Time PCR System in a 384-well transparent plate with a final reaction volume of 10 μl per well. The starting temperature of 25 °C was ramped up to 99 °C and then gradually reduced back to 25 °C. Ligands including ATP, AMP–PNP, ADP, AMP, and Sgv were added at a final concentration of 5 mM. Readouts were measured and analyzed with Protein Thermal Shift Software to obtain Boltzmann *T*_m_ values. At least three replicates of individual runs from the same preparation of protein were performed to obtain the mean and standard deviation of *T*_m_ values.

## Data availability

The coordinates and map coefficients of GRK5 structure were deposited in PDB with the accession codes 9CKO, 9CKP, 9CKQ, 9CKR, 9CKS, and 9MX2 as listed in Table S1. Other supporting data not shown are available from the corresponding author upon reasonable request (jtesmer@purdue.edu).

## Supporting information

This article contains supporting information (Figs. S1–S9).

## Conflict of interest

The authors declare that they have no conflicts of interest with the contents of this article.
