# Supplementary material for: Structural insights into the catalytic cycle of G protein–coupled receptor kinase 5 and a possible regulatory site for potassium ion
Source: J Biol Chem. 2025 May 29;301(7):110309. doi: 10.1016/j.jbc.2025.110309 (PMC12268638; doi:10.1016/j.jbc.2025.110309)
Supplement: Table S1 [file mmc1.docx]

**Table S1. Crystal collection and refinement statistics**

| **Data Collection** | **GRK5_WT_·Sgv** | **GRK5_D311N_·Sgv** | **GRK5_D311N_** | **GRK5_D311N_ ·ATP∙Mg^2+^** | **GRK5_D311N_ ·ATP∙Mn^2+^** | **GRK5_D311N_·Sgv (pH 6)** |
| --- | --- | --- | --- | --- | --- | --- |
| X-ray source | APS 23-ID-B | APS 23-ID-B | APS 23-ID-B | APS 23-ID-B | APS 23-ID-B | NSLS-II 17-ID-1 |
| Wavelength (Å) | 1.033 | 1.033 | 1.033 | 1.033 | 1.033 | 0.9201 |
| Resolution range (Å) | 29.5 - 2.8 (2.88 - 2.8) | 29.3 - 2.6 (2.69 - 2.6) | 24.3 - 2.7 (2.79 - 2.7) | 30.0 - 2.8 (2.88 - 2.8) | 28.8 - 3.08 (3.2 - 3.08) | 30.0 - 3.5  (3.68 - 3.5) |
| Space group | *P* 4_1_2_1_ 2 | *P* 4_1_2_1_ 2 | *P*4_1_2_1_ 2 | *P*4_1_2_1_ 2 | *P*4_1_2_1_ 2 | *P*4_1_2_1_ 2 |
| Unit cell | 140.3 140.3 78.8 | 140.2 140.2 77.8 | 137.3 137.3 71.0 | 136.6 136.6 70.6 | 137.8 137.8 71.3 | 140.3 140.3 78.0 |
| Total reflections | 308443 (26005) | 453678 (39913) | 337289 (31011) | 246928 (20589) | 177940 (19911) | 134818  (19646) |
| Unique reflections | 22966  (1868) | 33722  (3262) | 26003  (2306) | 18519  (1504) | 13233  (1442) | 8313  (1152) |
| Redundancy | 13.4 (13.9) | 13.5 (12.2) | 13.0 (13.4) | 13.3 (13.7) | 13.4 (13.8) | 16.2 (17.1) |
| Completeness (%) | 99.95 (100.00) | 99.90 (99.83) | 99.97 (100.00) | 99.97 (99.85) | 99.95 (99.93) | 92.39 (63.03) |
| Mean *<I>/<σ>* | 7.7 (0.4) | 9.6 (0.5) | 9.7 (0.4) | 9.6 (0.50) | 8.3 (0.75) | 4.46 (0.76) |
| R_merge_ | 0.247 (4) | 0.148 (3.3) | 0.144 (3.6) | 0.190 (3.7) | 0.251 (3.5) | 0.7059 (3.8) |
| R_meas_ | 0.257 (4.1) | 0.154 (3.5) | 0.150 (3.7) | 0.197 (3.8) | 0.26 (3.6) | 0.7288 (4.0) |
| R_pim_ | 0.0695 (1.1) | 0.0417 (0.97) | 0.0418 (1.0) | 0.0537 (1.04) | 0.0708 (0.96) | 0.1788 (0.96) |
| **Refinement** |  |  |  |  |  |  |
| R-work | 0.240 | 0.234 | 0.235 | 0.234 | 0.230 | 0.278 |
| R-free | 0.280 | 0.270 | 0.294 | 0.286 | 0.280 | 0.346 |
| Protein atoms | 4387 | 4372 | 4000 | 4078 | 4078 | 4372 |
| Ligand atoms | 37 | 26 | 0 | 43 | 43 | 26 |
| RMSD (bonds) | 0.004 | 0.005 | 0.003 | 0.002 | 0.001 | 0.002 |
| RMSD (angles) | 0.6 | 0.6 | 0.6 | 0.6 | 0.5 | 0.52 |
| Ramachandran favored (%) | 93.5 | 96.6 | 96.1 | 94.0 | 95.6 | 94.19 |
| Ramachandran outliers (%) | 0.19 | 0 | 0.41 | 0.6 | 0.2 | 0 |
| Clashscore | 5.9 | 4.6 | 4.6 | 7.7 | 1.5 | 1.71 |
| **B-factor** |  |  |  |  |  |  |
| Average  B-factor | 86.3 | 95.2 | 91.6 | 103 | 122 | 94.86 |
| protein | 86.6 | 95.7 | 91.7 | 103 | 122 | 94.98 |
| ligands | 72.3 | 70.7 | – | 157 | 168 | 76.41 |
| **PDB Code** | 9CKO | 9CKP | 9CKQ | 9CKR | 9CKS | 9MX2 |

RMSD, root mean square deviation. Residues in parentheses correspond to the highest resolution shell of data.
