# Supplementary material for: Structural insights into the catalytic cycle of G protein–coupled receptor kinase 5 and a possible regulatory site for potassium ion
Source: J Biol Chem. 2025 May 29;301(7):110309. doi: 10.1016/j.jbc.2025.110309 (PMC12268638; doi:10.1016/j.jbc.2025.110309)
Supplement: Table S2 [file mmc2.docx]

| Ligand | IC_50_ (no K_3_Cit) | IC_50_ (with K_3_Cit) | Fold-change | Substrate |
| --- | --- | --- | --- | --- |
| Sgv | 11 ± 3 μM (2) | 1.3 ± 0.7 μM (2) | 8 | tubulin |
| AMP | 0.7 ± 0.2 mM (2) | 0.46 ± 0.06 mM (2) | 1.5 |  |
| ADP | 28 ± 1 μM (2) | 11 ± 10 μM (2) | 3 |  |
| Sgv | 4.5 ± 0.5 μM (2) | 0.7 ± 0.6 μM (2) | 6 | Rho* |
| AMP | 1.0 ± 0.1 mM (2) | 0.5 ± 0.1 mM (2) | 2 |  |
| ADP | 56 ± 6 μM (2) | 39 ± 3 μM (2) | 1.5 |  |

**Table S2. Ligand inhibition of GRK5 activity in the presence of K_3_Cit.** IC_50_ values for GRK5 phosphorylation on tubulin or Rho* in ROS in presence of ligands (Sgv, AMP, ADP) without K_3_Cit, or with 2 mM K_3_Cit for tubulin phosphorylation or 6 mM K_3_Cit for Rho* phosphorylation, shown with mean and SD calculated using a 3-parameter dose response curves from two replicates. Dose response curves are shown in **Figure S3.**
