# Supplementary material for: Structural insights into the catalytic cycle of G protein–coupled receptor kinase 5 and a possible regulatory site for potassium ion
Source: J Biol Chem. 2025 May 29;301(7):110309. doi: 10.1016/j.jbc.2025.110309 (PMC12268638; doi:10.1016/j.jbc.2025.110309)
Supplement: Supporting information [file mmc3.docx]

**Supporting Information**

**Structural insights into the catalytic cycle of G protein-coupled receptor kinase 5 and a possible regulatory site for potassium ion**

**Authors**

Yueyi Chen, John J. G. Tesmer

Department of Biological Sciences, Purdue University, West Lafayette, IN 47907 (USA) and the Department of Medicinal Chemistry and Molecular Pharmacology

**Figure legends**

**Figure S1. SDS-PAGE gel of purified GRK5.** SDS-PAGE of GRK5_D311N_ elutions on a S200 size-exclusion chromatography compared to that of GRK5_WT_ which is heterogeneously phosphorylated and exhibits multiple bands.

**Figure S2. Lattice packing in Sgv-bound and ligand-free GRK5 structures.** Crystal lattice contacts within 4 Å from the unique chain are shown. **A)** Lattice packing in the structure of the GRK5·Sgv complex. The unique chain has the same color scheme as in **Fig. 1**. Purple symmetry mates pack against the RH domain, and cyan pack against the kinase domain. **B)** Lattice packing in the structure of ligand-free GRK5. An approximately 5° rotation centered from the large lobe away from the small lobe resulted in loss of density for αN’ and the AGC C-tail region. One additional lattice contact is formed in the ligand-free structure with the RH domain (lower symmetry mate). **C)** Low pH does not drive the conformational change from the structure in panels A to B. The lattice packing of GRK5·Sgv crystals soaked in 200 mM potassium citrate tribasic, 20% PEG3350 at pH 6 is the same as in in panel A.

**Figure S3. Structural comparison between GRK5-ATP with GRK1-ATP. A)** Overlay of GRK5 bound to ATP·Mg^2+^ (yellow) and GRK1 bound to ATP·2Mg^2+^ (3C4W, green), aligning the small lobe (residues 180 to 270). The large lobe exhibits a rotation away from the small lobe in GRK5 of ~9° compared to that of GRK1 primarily along PC2 axis (Fig. 3B). **B)** Close-up view of ATP binding site revealed that the phosphate/Mg^2+^-coordinating side chains (shown in sticks) are further away in the GRK5 structure.

**Figure S4. Dose-dependent GRK5 inhibition in presence of K_3_Cit, Na_3_Cit, KCl, and NaCl.** The gel images shown are representative autoradiogram phosphorimages for ^32^P-labeled products ***A)*** tubulin; ***B)*** Rho* in ROS; ***C)*** Rho* in LMNG:CHS under a specific salt concentration. Dose curves were plotted with normalized data in each replicate, using the no-salt condition as 100% and blank as 0%. Three replicates from the same preparation of protein are plotted with error bars reflecting SD.

**Figure S5. Inhibition of GRK5 by adenosine analogs in the presence or absence of K_3_Cit.** Dose-dependence of GRK5 activity towards **A)** tubulin or **B)** Rho* in presence of ligands (Sgv, AMP, ADP) with or without K_3_Cit. Two replicates from separate experiments using the same preparation of protein were plotted for mean and standard deviation of IC_50_ values.

**Figure S6. Changes in T_m_ of GRK5 upon addition of ADP/ATP and various salts**. T_m_ of GRK5_WT_ were measured under the same conditions as mentioned in Figure 6 with ligands ADP/ATP. **A)** Three or more replicates from separate experiments (circle) are shown along with their SD (box). **B)** Dose-dependent change in ΔT_m_ of GRK5 in the presence of ATP versus increasing concentrations of K_3_Cit is biphasic. This behavior is not replicated by Na_3_Cit suggesting that K^+^ is stabilizing a distinct conformation less able to bind ATP.

**Figure S7. Changes in T_m_ of GRK6 upon addition of Sgv ± salt**. T_m_ of GRK6 measured under the same conditions as in Figure 6. Three or more replicates from separate experiments (circles) are shown along with their SD (box). Note that differences in the Sgv-induced shift in T_m_ for GRK6 compared to GRK5 may partially reflect different basal T_m_ values in that both GRK2 and GRK6 have a higher T_m_ than GRK5 under basal conditions (**Figure S9**).

**Figure S8. Differences in T_M_ of GRK2 upon addition of ligands ± salt**. T_m_ of GRK2 measured in the same conditions as in Figure 6. Three or more replicates from separate experiments (circles) are shown along with their SD (box). Significance of GRK2 with Sgv alone were compared to those with added K_3_Cit using a one-tailed t-test, and the resulting P values shown in corresponding labels (ns: P > 0.05, *: P ≤ 0.05). Note that differences in the Sgv-induced shift in T_m_ for GRK2 compared to GRK5 may partially reflect their intrinsic basal T_m_ values. Both GRK2 and GRK6 have a higher T_m_ than GRK5 under these conditions (**Figure S9**).

**Figure S9. T_m_ of GRK2, GRK5, and GRK6 ± ligands**. T_m_ is shown as the mean ± SD (box) from three separate experiments shown in individual point (circle) using the same preparation of protein. Bars are color-coded as GRK-only in yellow, with ATP in blue, AMPPNP green, ADP purple, and Sgv orange.
